# Role of ferroptosis and ferroptosis-related long non'coding RNA in breast cancer

**DOI:** 10.1186/s11658-024-00560-2

**Published:** 2024-03-26

**Authors:** Shasha Xiang, Wen Yan, Xing Ren, Jianbo Feng, Xuyu Zu

**Affiliations:** 1 Cancer Research Institute, The First Affiliated Hospital, Hengyang Medical School, University of South China, Hengyang, 421001 Hunan China; 2 Department of Clinical Laboratory Medicine, The First Affiliated Hospital, Hengyang Medical School, University of South China, Hengyang, 421001 Hunan China

**Keywords:** LncRNA, Breast cancer, Ferroptosis, Ferroptosis-related lncRNA, Biomarker

## Abstract

Ferroptosis, a therapeutic strategy for tumours, is a regulated cell death characterised by the increased accumulation of iron-dependent lipid peroxides (LPO). Tumour-associated long non-coding RNAs (lncRNAs), when combined with traditional anti-cancer medicines or radiotherapy, can improve efficacy and decrease mortality in cancer. Investigating the role of ferroptosis-related lncRNAs may help strategise new therapeutic options for breast cancer (BC). Herein, we briefly discuss the genes and pathways of ferroptosis involved in iron and reactive oxygen species (ROS) metabolism, including the X_C_^−^/GSH/GPX4 system, ACSL4/LPCAT3/15-LOX and FSP1/CoQ10/NAD(P)H pathways, and investigate the correlation between ferroptosis and LncRNA in BC to determine possible biomarkers related to ferroptosis.

## Introduction

The GLOBOCAN 2020 Global Cancer Burden report states that the risk of breast cancer (BC) is higher in women, which is the fourth leading cause of cancer-related deaths worldwide [1]. BC, which can be classified as either invasive or non-invasive [2], is a condition in which various carcinogens act on the breast epithelial cells, resulting in abnormal or uncontrolled proliferation [3]. BC is typically characterised by the presence of breast lumps, nipple discharge and increased/enlarged lymph nodes in the axilla. In advanced stages, cancer cells can metastasise to distant locations, resulting in multi-organ damage and increasing the risk of mortality [4, 5].

Programmed cell death and accidental cell death (ACD) are the two main types of cell death. Accidental attacks and injuries can trigger ACD, outweighing any control mechanism, and are regulated by precise signalling cascades triggered by specific effector molecules with distinct biochemical, functional and immunological effects [6]. The different forms of regulated cell death include apoptosis, necrosis, autophagy and ferroptosis. This study explored ferroptosis as a novel regulatory mode of cell death dependent on iron and lipotoxicity [6, 7], and several genes and pathways are involved in the modulation of iron and reactive oxygen species (ROS) metabolism, including the X_C_^−^/GSH/GPX4 system and ACSL4/LPCAT3/15-LOX and FSP1/CoQ10/NAD(P)H pathways [8–10]. Dysregulation of iron metabolism, one of the risk factors for tumours, and the overdependence of cancer cells on iron proliferation promote the growth of tumour cells [11]. Activating ferroptosis in cancer cells is a novel approach to mitigating cancer risk, particularly for those resistant to conventional chemotherapy [12, 13]. Cilamethicin and lapatinib can trigger ferroptosis in BC cells, suggesting that they could serve as viable treatments for BC patients [14]. Nevertheless, studies analysing the relationship between BC and ferroptosis are lacking, and the influence of ferroptosis on the prognosis of patients with BC remains uncertain.

Long non-coding RNAs (lncRNAs) are RNA molecules that are longer than 200 nucleotides and lack the ability to encode proteins [15, 16]. Because of their unique involvement in cancer, lncRNAs have gained considerable attention [17]. Peptides or proteins encoded by tumour-associated lncRNAs increase efficacy and reduce mortality in combination with conventional cancer drugs and radiotherapy [18]. LncRNA-encoded ASRPS contributes to the progression of triple-negative breast cancer (TNBC), whereas the lncRNA HOXB cluster anti-sense RNA 3 (HOXB-AS3) peptide inhibits the growth of colorectal cancer (CRC) [19, 20]. Moreover, lncRNAs regulate ferroptosis in BC [21]. However, existing studies on lncRNAs related to ferroptosis in BC are scarce, and only a few lncRNAs involved in regulating ferroptosis have been identified. However, the recognition of the repetitive molecular mechanisms of lncRNAs has been made possible by emerging technologies that have enhanced the ability of researchers to functionally annotate cancer-related lncRNAs, such as the identification of potential ferroptosis-associated lncRNAs, which can be achieved using high-speed sequencing technologies [22].

This review discusses lncRNAs associated with the activation or inhibition of cellular ferroptosis, which exert anti-cancer effects, thereby providing potential insights for strategising new cancer treatment regimens. However, studies on ferroptosis-related lncRNAs in BC remain limited. Therefore, we briefly discuss the role of ferroptosis in BC, the association between BC and lncRNAs,and identify potential ferroptosis-related lncRNAs in BC.

## Ferroptosis: a brief overview

**Fig. 1:**
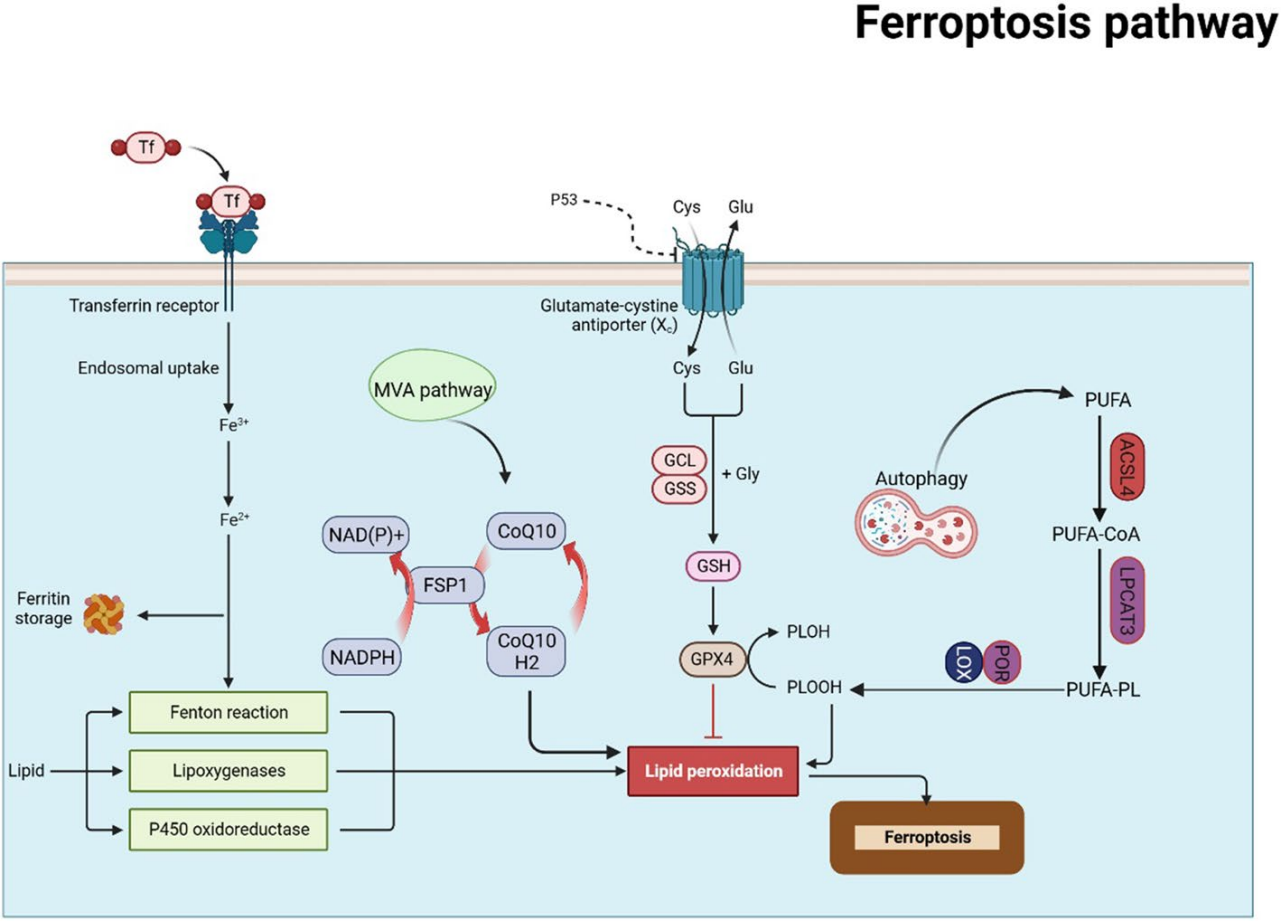
Ferroptosis pathways. Genes and pathways of ferroptosis involved in the regulation of iron and ROS metabolism, including the system X_C_^−^/GSH/GPX4, and ACSL4/LPCAT3/15-LOX and FSP1/CoQ10/NAD(P)H pathways. Created with https://www.biorender.com/

Ferroptosis was first discovered in 2003 and involved the use of erastin to selectively induce cell death in genetically engineered cells with oncogenic RAS mutations, but not in normal cells [23]. Brent Stockwell, in 2012, coined the term ferroptosis for the iron-dependent cell death mode of non-apoptotic RCD induced by erastin [24]. Ferroptosis is a distinct type of regulated cell death mediated by iron and lipotoxicity that has been recently identified. Ferroptosis inhibits the activity of the lipid repair enzyme glutathione peroxidase 4 (GPX4), leading to the accumulation of lipid ROS, particularly lipid hydroperoxides [7]. In terms of genetics, multiple genes regulate ferroptosis. In contrast to other morphological changes associated with cell death, ferroptosis occurs primarily within the cell. This results in smaller mitochondria, increased membrane density, decreased and disappeared cristae, fragmentation of the outer membrane without disruption of the cell membrane and minimal transformation in the morphology of the nucleus without chromatin concentration [24–26]. Biochemically, the phospholipid peroxidase GPX4 primarily contributes to the deficiency in peroxidation repair capacity, acquisition of reactive iron and oxidation of polyunsaturated fatty acids (PUFA)-containing phospholipids that induce ferroptosis [27]. The intracellular antioxidant capacity decreases and lipid ROS accumulates, ultimately leading to cellular ferroptosis. Glutathione peroxidase is affected by various pathways, such as the X_C_^−^/GSH/GPX4 system, and the ACSL4/LPCAT3/15-LOX and FSP1/CoQ10/NAD(P)H pathways [8–10] (Fig. 1).

### System X_C_^−^/GSH/GPX4 and ferroptosis

Both impaired elimination and overproduction of lipid peroxide (LPO) during ferroptosis can lead to its accumulation to lethal levels. Cystine availability, glutathione (GSH) biosynthesis and GPX4 function are required to maintain redox homeostasis and protect cells from ferroptosis [24, 28, 29].

System X_C_^−^/GSH/GPX4 is the antioxidant system that is crucial for ferroptosis [30]. System X_C_^−^ functions as a glutamate–cysteine reverse transporter at the plasma membrane, importing cysteine into the cytosol to facilitate GSH biosynthesis [31]. The inhibition of system X_C_^−^ results in a reduction in the intracellular cysteine pool, which is a precursor for glutathione synthesis [32]. GPX4 is the primary enzyme involved in the reduction and detoxification of phospholipid hydroperoxides (PLOOHs) in mammalian cells [33]; therefore, a general mechanism for the induction of ferroptosis by erastin/RSL3 has been identified. GSH peroxidase 4 inhibitor (RSL3) directly inactivates GPX4, whereas erastin indirectly inactivates it by inhibiting cysteine input, thereby depriving the cells of cysteine, an essential cellular component of GSH. Therefore, the accumulation of PLOOHs may cause rapid and irreversible damage to cell membranes, resulting in cell death.

GPX4 converts GSH to oxidised glutathione disulphide (GSSG), reduces LPO and maintains cell redox homeostasis [28]. Moreover, GPX4 is the only enzyme that directly reduces hydrogen peroxide from biofilm lipids [34]. Suppressing the system X_C_^−^/GSH/GPX4 axis results in the accumulation of LPO, thereby leading to ferroptosis; for example, system X_C_^−^ activity is directly inhibited by the ferroptosis inducer erastin, which disrupts redox homeostasis and increases LPO accumulation, leading to ferroptosis [24]. Intracellular and extracellular cysteine are needed to maintain glutathione biosynthesis and inhibit mammalian cell death, which can also be treated with iron sequestrants or hydrophilic antioxidants [35].

### ACSL4/LPCAT3/15-LOX with ferroptosis

Clustered regularly interspaced palindromic repeats (CRISPR)–Cas9 and genome-wide haploid-based screening analyses have identified two membrane turnover enzymes: lysophosphatidylcholine acyltransferase 3 (LPCAT3) and acyl-coenzyme A synthase long chain family member 4 (ACSL4) [36, 37] as key drivers of ferroptosis. These enzymes are essential for endogenous iron chain activation through metabolic lipid reprogramming [38]. ACSL4 is a prominent isoenzyme involved in the biometabolism of PUFAs and determines their susceptibility to ferroptosis [39]. Lipid synthesis-mediated production of PUFAs increases the susceptibility to ferroptosis [40]. The entry of PUFAs into phospholipids, a crucial step in ferroptosis, requires ACSL4 [40], which links coenzyme A to long-chain PUFAs, which are then transesterified into phospholipids by several LPCATases, increasing the integration of long-chain PUFAs into lipids and membranes [7].

Elevated ACSL4 expression increases the sensitivity of cells to ferroptosis by optimising the catalysis of several PUFAs, with a strong affinity for arachidonic acid (AA) and adrenaline (AdA). ACSL4 catalyses the conversion of AA and AdA into AACoA and AdA-CoA, respectively, resulting in LPO production. The derivatives were first esterified with LPCAT3 to form phosphatidylethanolamines (AA-PE and AdA-PE), followed by the direct oxidation of their lipid hydrogen peroxide by 15-LOX (ALOX15), which acts as a signal for ferric ions and, ultimately, promotes ferroptosis [31, 36, 41, 42]. Additionally, this process affects the cellular lipid composition [8, 36]. Therefore, ACSL4/LPCAT3/15-LOX may play an important role in the generation of lethal LPOs during ferroptosis.

ACSL4 determines the susceptibility to ferroptosis by modifying cellular lipid composition [36, 43]. The lipoxygenase enzyme, which contains iron, promotes cell death by producing LPO via lipid biosynthesis in ACSL4 [40]. ACSL4, a target of miR-424-5P, is upregulated in ovarian cancer (OC) and inhibits OC cell ferroptosis [44]. The inhibition of ACSL4 expression may be the primary mechanism that renders cells insensitive to iron leaps.

### FSP1/CoQ10/NAD(P)H with ferroptosis

Apoptosis-inducing factor mitochondria-associated 2 (AIFM2), a member of the apoptosis-inducing factor (AIF) family, is involved in oxidoreductase function and can induce programmed cell death [45]. Recently, AIFM2 was recognised as an anti-iron porphyrin gene and was later renamed ferrocyte apoptosis suppressor protein 1 (FSP1). FSP1 inhibits iron through ubiquitin ketone (CoQ10), a reductant that scavenges the lipid peroxyl radicals responsible for lipid peroxidation. The use of FSP1 as a pharmacological target in combination with GPX4 inhibitors induces ferroptosis in various tumour types [46].

CoQ10 plays key roles in the mevalonate (MVA) pathway; regulating the MVA pathway could be a possible strategy for controlling the course of ferroptosis [47]. Following cardamoylation, FSP1 is recruited to the plasma membrane, where it acts as an oxidoreductase to catalyse the generation of ubiquitin from CoQ10 via NADPH. As a lipophilic anti-radical catcher, NADPH decreases LPO levels [46, 48]. Therefore, FSP1/CoQ10/NAD(P)H acts synergistically with GPX4 and GSH to protect against phospholipid peroxidation and ferroptosis [46].

### Other genes and pathways for ferroptosis

P53 mediates cell cycle pausing, senescence and apoptosis, and its inactivation is a key factor in the formation of most tumours; therefore, the p53 gene is considered a potential tumour suppressor gene. Additionally, P53 is involved in various metabolic activities [49]. P53 downregulates the expression of solute carrier family 7 member 11 (SLC7A11) and inhibits the systemic uptake of cystine through GPX4 activity. This leads to reduced cellular antioxidant capacity and the accumulation of lipid ROS, resulting in ferroptosis [49, 50].

In addition, autophagy contributes to ferroptosis. Although ferroptosis can lead to the lipid peroxidation of plasma membranes, the major membrane modulator proteins remain unclear [51, 52]. Autophagy removes a wide range of components by forming dynamic membrane structures, such as phagosomes, autophagic vesicles and autophagosomes [53]. However, excessive autophagic activity mediates ferroptosis [54]. Autophagy facilitates swift, non-apoptotic, non-necrotic cell death during amino acid starvation. This condition triggers potent autophagy, but only if sufficient serum is provided in the culture medium, as it requires iron-supporting transferrin and the amino acid glutamine in saline. Death is triggered by cysteine deficiency in the cell growth media, which could be attributed to ferroptosis [55]. Autophagy is implicated in cysteine deprivation and is sensitised to ferroptosis through the autophagic degradation of ferritin, which is also termed ferritin autophagy, leading to increased levels of unstable iron in cells [56, 57]. For instance, HPCAL1 (hippocampalin-like protein 1) is a novel autophagic receptor that selectively degrades CDH2 (calbindin 2) during ferroptosis. HPCAL1 facilitates ferroptosis through its non-canonical role in autophagy. The CDH2/*N*-calbindin protein is a straightforward substrate for HPCAL1-dependent autophagic degradation and triggers ferroptosis by compromising membrane tension [52, 58].

In addition, methods for inducing or inhibiting ferroptosis have been extensively studied. For instance, glucose starvation inhibits ferroptosis [59, 60], whereas arachidonic acid enhances RSL3-mediated ferroptosis in mouse foetal fibroblasts[38]. Other signalling pathways that regulate cellular ferroptosis, such as Keleh-like ECH-associated protein 1 (Keap1), nuclear factor red lineage-associated factor 2 (Nrf2) and lymphoid tissue-specific deconjugating enzyme (LSH), have also been identified. Furthermore, EgI nine homologue 1 (EGLN1)/cellular myeloid cell tumour proto-oncogene (c-Myc), sulphur transfer, mucin 1C-terminal (MUC1-C)/ systemic X_C_^−^ (xCT) and heat-shock factor-1 (HSF1)/heat-shock protein beta 1 (HSPB1) pathways mediate ferroptosis [61].

## Association between ferroptosis and BC

Ferroptosis has been implicated in the pathological processes of several disorders, such as neurological disorders, blood disorders, kidney damage, ischaemia–reperfusion injury and tumours. However, the natural mechanisms underlying the induction of ferroptosis under these conditions remain unclear. Ferroptosis is an oxidative stress-induced cell death process that is closely associated with cellular metabolism. Cancer cells, which have a more active metabolism and a higher ROS load, may have a stronger tendency towards ferroptosis. Because cellular ferroptosis inhibits tumour growth, targeting ferroptosis pathways could be a promising anti-cancer strategy [27]. In addition, ferroptosis occurs during cancer treatment. For example, low-density lipoprotein (LDL)–docosahexaenoic acid (DHA) nanoparticles and sorafenib induce ferroptosis in hepatocellular carcinoma cells [9, 62]. Cysteine dioxygenase 1 (CDO1) modulates erastin in vitro in gastric cancer cells [63], whereas cisplatin and dipeptidyl peptidase-4 (DPP4) regulate erastin-induced ferroptosis in colorectal cancer [64, 65]. Piperonylamine (PL), cyclophosphamide (CNA), liuzasulfapyridine combination, cottonin A (CN-A), phenethyl isothiocyanate (PEITC) and artesunate (ART) induce ROS generation, activation and ferroptosis in pancreatic cancer cell lines to inhibit proliferation [66, 67]. Erastin upregulates and activates P53, inhibits the activity of SLC7A11 and induces ferroptosis in lung cancer cells [68].

Cellular ferroptosis is recognised as a key mechanism by which certain chemotherapeutic agents induce cell death in cancer cells [65, 69]. BC is a diverse tumour; based on the hormone receptors (ER and PR) and HER2 (ERBB2) signatures, BC is clinically classified into three main subtypes: TNBC, tubular ER^+^ and PR^+^, and HER2^+^ [3, 70, 71]. The National Comprehensive Cancer Network (NCCN) recommendations recommends endocrine therapy for ER^+^ BC and anti-HER2-targeted therapy for HER2^+^ BC. Targeted therapies for TNBC are currently lacking [72]. Although the potential benefits of inducing ferroptosis in tumour therapy have been suggested, the genes related to ferroptosis have not been extensively studied in BC patients [73].

Treatment of TNBC remains challenging, and identifying the coordinated role of pathways in regulating ferroptosis will provide a fresh impetus for a therapeutic strategy for TNBC. TNBC was more sensitive to ferroptosis than ER^+^ BC [36]. TNBC can be divided into four categories: mesenchymal-like subtype (MES), luminal androgen receptor (LAR) subtype, immunomodulatory subtype (IM) and basal-like immunosuppressive subtype (BLIS) subtypes. The LAR subtype can induce ferroptosis using GPX4 inhibitors. This subtype is characterised by the upregulation of the oxidised phosphatidylethanolamine and glutathione metabolism (in particular, GPX4). Furthermore, inhibition of GPX4 not only leads to tumour ferroptosis but also enhances anti-tumour immune function [74]. By signalling epithelial-to-mesenchymal transition, MES cells can promote the activity of iron-connected molecules (as transferrin receptor 1, ferritin) and enhance iron uptake, storage and utilisation [10, 75]. Notably, this process is not limited to cancer cells but can also occur in non-cancerous cells. MES is characterised by an MES state enriched in iron metabolic pathways but lacking fatty acid (FA) metabolism and ROS pathway activity, indicating that, compared with LAR, MES subtypes are more susceptible to ferroptosis [76, 77]. Both IM and BLIS are characterised by typical stromal-like tumours in the presence of ferroptotic area [74].

One finding demonstrated that GPX4 expression was lower in BC MCF7 and MDA-MB-231 cell lines than in non-BC MCF10A cell lines [78], and that GPX4 expression was positively correlated with ER and PR labelling [79]. GPX4 may exert anti-tumour activity and reflect an improved differentiation phenotype in BC [73]. Erastin targets MDA-MB-231 cells selectively and effectively induces ferroptosis in TNBC cells [80].

Notably, ACSL4 expression levels in a subpopulation of TNBC cell lines were correlated with their sensitivity to ferroptosis reagents. This correlation appears to be similar to that observed in the treatment of refractory mesenchymal carcinoma cells and clear cell renal carcinoma cells [7]. ACSL4 is elevated in BC tissues compared with in healthy tissues adjacent to the cancer, and ACSL4 expression is negatively correlated with ER [81, 82]. Clinically, radiotherapy upregulates the expression of ACSL4, resulting in increased lipid synthesis and, consequently, oxidative injury, leading to ferroptosis [83]. High expression of ACSL4 promotes BC aggressiveness, is a potential prognostic indicator and therapeutic target [82], and plays a substantial role in radiation resistance in BC by modulating the expression of transporter proteins implicated in cancer resistance via the mTOR pathway and regulating forkhead box protein M1 (FOXM1) [84].

These findings indicate that ferroptosis may be an essential adaptation process for eradicating cancer cells [85].

## Role of lncRNAs in BC

LncRNAs are RNA molecules that are ˃ 200 nucleotides long and lack the ability to encode proteins [86]. They are widely present in humans and are critical in regulating human gene expression and physiological and pathological processes [87]. LncRNAs can be broadly classified into the following three types: direct linking to DNA or transcription factors at the transcription level; binding of mRNAs, miRNAs or proteins to modulate their activity and steady state in a post-transcriptional manner; and interference with the chromatin complex to activate or suppress gene expression in an epigenetic manner [86, 88–90].

The mammalian genome contains numerous lncRNAs. A small but increasing number of these lncRNAs have functional profiles in various processes and diseases, such as infection, innate immunity and acquired immunity [91–96]. Cancer is a genetic disease that involves an alteration in the flow of information within the cell to alter cellular homeostasis and promote growth [22]. Non-coding RNAs regulate inter- and intracellular signalling in BC [97]. LncRNA-encoded peptides affect BC cells [98]. For instance, the micropeptide CIP2A-binding peptide (CIP2A-BP) encoded by LINC0665 is highly correlated with the survival of BC recipients. Poor CIP2A-BP expression is associated with low survival in BC patients. In addition, CIP2A-BP levels in patients with metastatic BC were markedly lower than in those without metastasis. Both the introduction of the CIP2A-BP gene and direct infusion of the CIP2A-BP micropeptide markedly attenuated lung metastasis and improved overall survival, suggesting that the micropeptide CIP2A-BP suppressed the migration and invasion of TNBC cells [99]. LINC00908 encodes ASRPS, a potential anti-cancer micropeptide that is endogenously expressed and downregulated in TNBC and inhibits tumour angiogenesis in BC [19].

Several lncRNAs that promote BC development have been identified and their functions have been investigated. This information can aid in the diagnosis, prognostic judgement, pathogenesis prediction and therapeutic intervention for BC (Table 1).

**Table 1:** Role of various lncRNAs in breast cancer

LncRNA	Regulation	Work	References
HOTAIR	–	Potential metastatic, drug-resistant and prognostic regulators of BC; highly predictive of metastatic disease progression and overall survival	[123, 127]
	miR-206	Enhances BC cell proliferation	[129]
	Chondroitin sulfate	Enhances BC cell invasion	[130]
	miR-203/CAV1 axis	Influences BC cell migration, proliferation and invasion	[104]
	miR-20a-5p/HMGA2 axis	Influences BC cell apoptosis, growth, migration and invasion	[131]
	miR-129-5p/FZD7 axis	Promotes BC	[125]
UCA1	miR-375	Inhibits BC progression	[132]
RP11-19E11	E2F1	Proliferation and survival of basal BC	[133]
LINC00963	miR-324-3p/ ACK1	Promotes tumourigenesis and radiation resistance in BC	[134]
LINC00899	miR-425	Inhibits BC cell migration, proliferation and invasion	[135]
LINC01787	niR-125b	Promotes BC cell growth, proliferation and migration of BC xenografts	[136]
NKILA	IκB	Inhibits BC metastasis	[137]
Gas5	–	Sensitizes BC cells to ionising radiation by inhibiting DNA repair	[138]
NORAD	PUM1/Eif2 axis	Inhibits BC progression	[139]
	TGFβ	High expression is indicative of poor prognosis	[140]
	YAP pathway	Inhibits BC metastasis	[141]
BCRT1	miR-1303/PTBP3 axis	Promotes BC progression	[142]
SEMA3B-AS1	miR-3940/KLLN axis	Inhibits BC progression	[143]
NR2F1-AS1	IGF-1/IGF-1R/ERK pathway	Promotes angiogenesis in BC	[144]
BC069792	KCNQ4	Inhibits tumour progression in BC	[145]
SNHG1	macrophage M2-like polarization	Promotes BC growth and metastasis	[146]
GHET1	EMT	Promotes BC cell proliferation, invasion and migration	[111]
PRNCR1	microRNA-377/CCND2/MEK/MAPK axis	Promotes BC proliferation and inhibit apoptosis	[147]
NEAT1	miR-133b	Promotes migration and invasion of BC cells	[148]
NEF	–	Downregulated expression is suggestive of poor prognosis	[149]
TPA	TGFβ	Promotes BC invasion and metastasis	[150]
ERINA	E2F1/RB1 pathway	Inhibits cell-cycle progression and tumour cell proliferation	[151]
LCPAT1	MFAP2	Promotes BC progression	[152]
PlncRNA-1	TGFβ1, PHGDH	Inhibits the growth of BC	[153]
ITGB2-AS1	ITGB2	Promotes BC migration and invasion	[154]
RP1-5O6.5	KLF5	Promotes growth and metastasis of BC	[155]
LSINCT5	–	Promotes BC cell proliferation	[105]
LncRNA-CDC6	microRNA-251	Promotes BC progression and function as ceRNA	[156]
MALAT1	miR-497-5p/SHOC2 axis	Regulates the paclitaxel resistance of BC	[157]
	–	Overexpression inhibits BC metastasis in transgenic, xenograft and homologous models	[158]
Uc003xsl.1	NFκB/IL8 axis	Promotes progression of TNBC, growth and metastasis	[159]
CARMN	miR143-3p	Promotes prognosis and chemosensitivity of TNBC	[160, 161]
BREA2	Notch signalling	Drivers of metastasis in BC	[162]
DIO3OS	PTBP1, LDHA	Correlated with a worse prognosis in BC patients on AI therapies	[163]
KB-1980E6.3	lncRNA KB-1980E6.3/IGF2BP1/c-Myc axis	Maintain the stemness of BC stem cells	[164]
BORG	TRIM28, BORG	Drives BC metastasis and disease recurrence; elicits the metastatic outgrowth of latent BC cells	[165]
EPB41L4A-AS1	–	Regulates cell metastasis, proliferation and apoptosis in BC	[166]
FOXD3-AS1	miR-127-3p	Affects BC cell proliferation, migration, invasion and growth	[167]
LGALS8-AS1	miR-125b-5p/SOX12	Promotes BC metastasis	[168]
CASC15	miR-654-5p/MEF2D axis	Regulates BC cell stemness	[169]
GHET1	–	Knockdown suppresses BC activity	[170]
DUXAP8	PI3K/AKT/mTOR pathway, EZH2-E-cadherin/ RHOB axis	Promotes radiation resistance in BC	[171]
RP11-214F16.8	SENP3	Drives BC tumourigenesis	[172]
MIR17HG	miR-454-3p	Suppresses BC cell proliferation and migration	[173]
EGOT	Hedgehog pathway	Decreases BC cell viability and migration	[174]
SNHG6	miR-26a/VASP axis	Silencing suppresses proliferation and invasion of BC cells	[175]
SNHG8	miR-634/ZBTB20 axis	Serves as an oncogene in BC	[176]
PVT1	miR-145-5p	Influences glycolysis in BC cells	[177]
APOC1P1-3	miRNA-188-3p	Promotes metastasis in BC	[178]
FGD5-AS1	has-miR-195-5p/NUAK2 axis	Promotes BC progression	[179]
FBXL19-AS1	miR-718	Promotes proliferation and invasion of BC cells	[180]
PTCSC3	lncRNA H19	Inhibits TNBC cell proliferation	[181]

### LncRNAs that inhibit BC development

NKILA, NEF,GAS5, MT1JP, LET, LncKLHDC7B and TFAP2A-AS1 prevent BC cell invasion and migration; NLIPMT, XIXT, MALAT1 and MEG3 inhibit distant metastasis in BC cells [100].

LINC02273 knockdown inhibits BC metastasis [101]. LncRNA GAS5 is frequently downregulated in several cancers. In BC, GAS5 activates several proteins, including DKK2, PTEN, SUFU, PDCD4 and FOXO1, via various miRNA-mediated competing endogenouse RNA (ceRNA) mechanisms. These mechanisms involve miR-196a-5p, miR-21, miR-221-3p, miR-222 and miR-378a-5p, which bind to multiple microRNA response elements (MREs) in GAS5 to upregulate the expression of BC suppressor proteins. Furthermore, through epigenetic and other mechanisms, GAS5 may enhance the sensitivity to several drugs and improve prognosis [102].

### LncRNAs that promote BC development

Several lncRNAs that affect the invasiveness, proliferation and apoptosis of BC cells have been identified. For instance, LINC00461, DANCR, H19, HOX transcriptional anti-toxic intergenic RNAs (HOTAIR), LINC00152, LINC01857 and NEAT1 facilitate BC cell invasion and migration; and HOTAIR, H19, MALAT1, RP1 and HIF1A-AS2 promote BC cell long-distant metastasis. LncRNAs, including H19 [103], PRNCR1, HOTAIR [104], LSINCT5 [105], SRA [106], Smad7 [107], NEAT1 [108], LINC01296 [109] AFAP1-AS1 [110], GHET1 [111], BRAF [112] and SNHG12 [113] promote cell proliferation and inhibit apoptosis in BC. Some lncRNAs can promote BC cell resistance, such as UCA1 [114], CRALA [115], lnc-ATB [116], LINC00518 [117] and DSCAM-AS1 [118].

H19 is located in the human genome downstream of IGF2, and its levels are elevated in a variety of cancers, notably BC, promoting BC cell proliferation [103, 119]. H19 expression is significantly upregulated in tamoxifen-refractory BC cell lines and tissues, and silencing of H19 in MCF7/TAMR cells is sensitive to tamoxifen therapy in vivo and in vitro [120]. Metformin may cause ferroptosis in BC by blocking autophagy in H19 [121]. UCA1 inhibits p27, which partially contributes to its oncogenic role in BC. Overexpression of UCA1 is overexpressed causes hnRNP I in the cytoplasm to be recruited to UCA1, reducing the access of p27 to hnRNP I, inducing a cell cycle pause in the G1 stage; therefore, UCA1 could be a potential biomarker for BC diagnosis [122].

HOTAIR, a new type of lncRNAs belonging to a subclass of intergenic lncRNAs tightly regulates genes related to mammalian embryonic development [123]. HOTAIR expression is highly upregulated in BC, and silencing HOTAIR induces apoptosis and prevents cell proliferation. The mechanism of action involves linking miRNA and post-transcriptional networks to promote BC development [123, 124]. For instance, HOTAIR acts as a mediator between frizzled homologue 7 (FZD7) and miR-129-5p, and promotes epithelial–mesenchymal transition and metastasis, leading to BC progression. HOTAIR knockdown inhibited tumour growth in a xenograft mode, whereas killing of miR-129-5p reversed the silencing function of HOTAIR and FZD7 restored the suppressive function of miR-129-5p, suggesting that HOTAIR controls the miR-129-5p/FZD7 axis [125]. HOTAIR further facilitates BC metabolism by targeting miR-601 via a sponge mechanism to control AKT signalling, which is dependent on zinc finger E-box binding homology box 1 (ZEB1) [126]. HOTAIR overexpression in surgically resected early stage BC is a strong predictor of metastatic disease progression and overall survival [127].

The significance of cell signalling pathways in tumourigenesis, tumour progression and metastasis cannot be overlooked. LncRNAs affect aspects of tumourigenesis by participating in or interfering with these pathways and, consequently, they exhibit either an oncogenic or a tumour-suppressive role [128].

## Ferroptosis-associated lncRNAs in BC

Diverse physiological conditions and pathological stressors trigger ferroptosis in humans and animals [24]. Ferroptosis has been increasingly recognised as an adaptive feature in the elimination of malignant tumours. The immune system plays a crucial role in the suppression of tumourigenesis, by removing cells that have been damaged by infection, environmental stress or a lack of key nutrients [182]. The classical oxidative stress pathway is an important therapeutic element that may contribute to ferroptosis. Despite the delicate balance between thiols and catalytic iron in cancer cells under sustained oxidative stress, this process occurs infrequently during cancer progression. However, the underlying molecular mechanisms remain unclear [13].

Abnormally expressed lncRNAs typically affect disease progression by regulating transcription and translation and can also influence cancer progression through the regulation of ferroptosis. The cytoplasmic lncRNA P53RRA in lung adenocarcinoma cancer cells binds to the structural portion of the Ras GTPase-activating protein-binding protein 1 (G3BP1) RNA recognition motif (RRM), leading to nuclear segregation of P53 and retained P53 in the nucleus and accumulation of lipid ROS in the nucleus, subsequently leading to cellular ferroptosis [21]. LncRNA LINC00618-induced ferroptosis increases lipid ROS and iron levels, and lowers SLC7A11 expression [183]. LncRNA GABPB1-AS1 modulates erastin-induced GABPB1 ferroptosis in HepG2 hepatoma cells [184]. Similarly, the GSK3β/Nrf2 signalling pathway is implicated in BC, which increases Nrf2 expression to counteract ferroptosis [185]. In addition, prominence protein 2 (PROM2) could reduce ferroptosis in BC cells and facilitate tumour progression by encouraging iron transport [186]. Because only specific lncRNAs are associated with ferroptosis, existing studies on ferroptosis-associated lncRNAs in BC are scarce.

RNA-sequencing data and one-way COX regression analyses in BC patients have led to the identification of 231 lncRNAs which affects the prognosis; 293 genes associated with ferroptosis were also downloaded from the Ferroptosis Database [187]. Furthermore, 11 lncRNAs (AC092916.1, L133467.1, USP30-AS1, AC108474.1, LINC01235, AL365356.1, AC072039.2, AC012213.3, LIPE-AS1, MAPT-AS1 and TDRKHAS1) that were significantly different were identified. Among them, lncRNA USP30-AS1 was co-expressed with nine ferroptosis-linked genes (SOCS1, CAPG, IFNG, PML, TNFAIP3, NCF2, SLC2A6, GCH1 and CYBB), suggesting that overexpression of USP30-AS1 in BC is associated with prolonged overall survival. LncRNA LIPE-AS1 interacts with five ferroptosis genes (GPX4, PHKG2, EGLN2, MAPK14 and HRAS), and improves the prognosis of BC patients. AC108474.1 interacts with five ferroptosis-related genes (HIC1, ISCU, PLIN4, CAV1 and TAZ), suggesting that AC108474.1 is also a protective factor for BC patients [188].

**Fig. 2:**
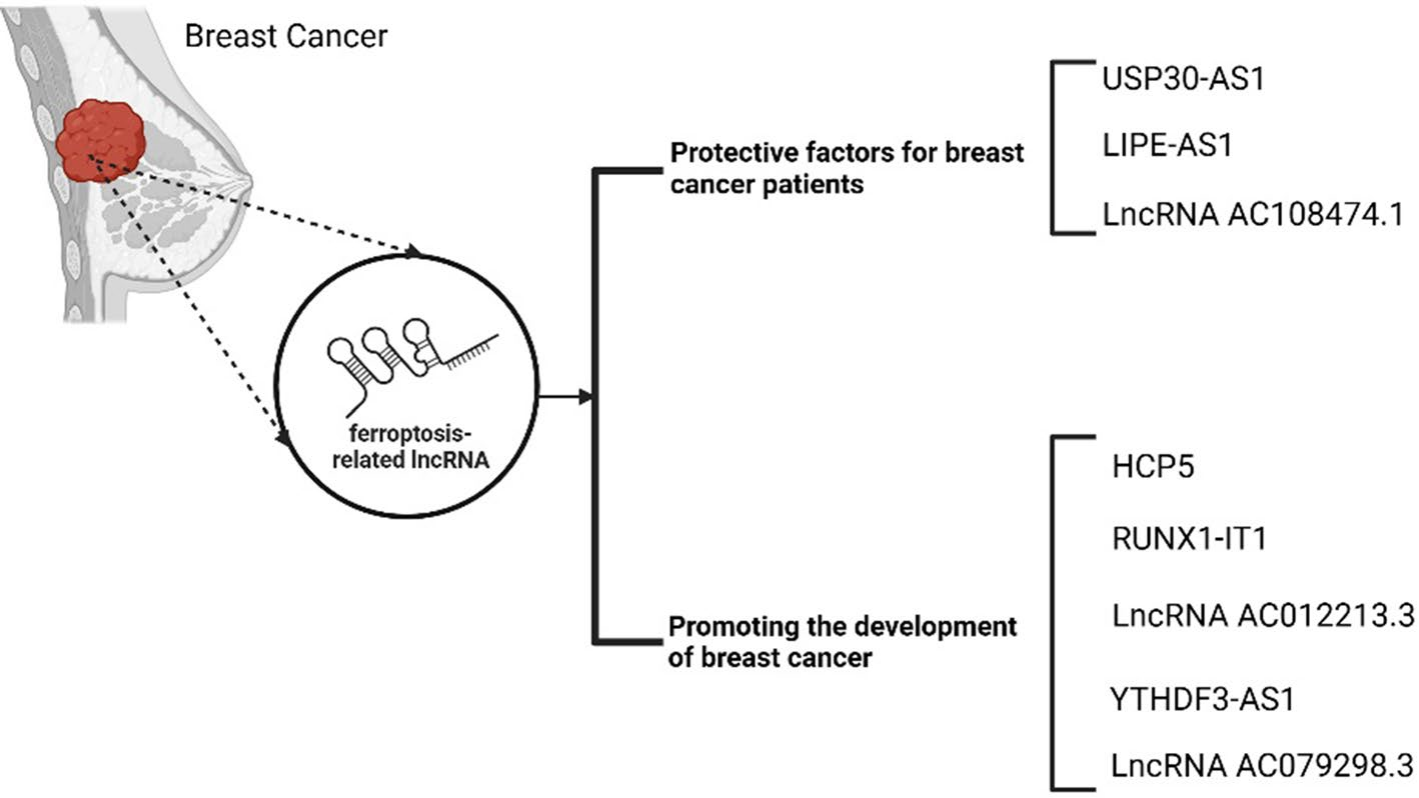
Ferroptosis-related lncRNAs in breast cancer. Created with https://www.biorender.com/

LncRNA HCP5 regulates baculoviral IAP repeat-containing 3 (BIRC3) by sponging miR-219a-5p as a ceRNA and promotes TNBC progression [189]. Moreover, the amino acid encoded by HCP5, HCP5-132aa promotes the malignant progression of TNBC through its dependence on GPX4 and lipid ROS levels. RNA sequencing results showed that silencing of the HCP5-132 amino acid (aa) open reading frame (ORF) resulted in an enrichment of differentially expressed genes (DEGS) associated with the ferroptosis pathway (which had a positive impact on intracellular Fe homeostasis, progesterone metabolic processes and cell proliferation), suggesting that disturbances in ferroptosis, progesterone metabolism and cell proliferation may affect BC development [190]. This resulted in an increase in the mitochondrial membrane density and a reduction in the mitochondrial cristae, with effects similar to those of erastin. In contrast, overexpression of HCP5-132aa ORF inhibited erastin-induced changes in mitochondrial morphology. Moreover, silencing of HCP5-132aa, along with the elevation of ROS levels when cells were primed with the ferroptosis enhancers RSL3 and erastin, was also counteracted by ferroptosis inhibitors and upregulation of HCP5-132aa. Furthermore, excessive expression of HCP5-132aa was associated with a worse patient prognosis, suggesting that HCP5-132aa might be a prognostic factor in TNBC [191] (see Fig. 2).

Knockdown of RUNX1 intronic transcript 1 (RUNX1-IT1), a newly identified lncRNA that plays a key role in breast carcinogenesis, was significantly overexpressed in human BC tissues, inhibited BC cell survival and invasion, and suppressed tumour growth in an in situ transplantation model. Furthermore, RUNX1-IT1 inhibited ferroptosis by increasing GPX4 expression. RUNXI-IT1 specifically binds directly to the *N*6-methyladenosine M6A reader, IGF2BP1, and promotes the assembly of the IGF2BP1 liquid–liquid phase (LLP) biomolecule condensate site, resulting in IGF2BP1 greater occupancy of GPX4 mRNA and increased GPX4 mRNA stability. The elevated GPX4 expression prevent lipid peroxidation and ferroptosis, thereby promoting BC development, which indicates that the abnormal regulation of RUNX1-IT1/IGF2BP1/GPX4 is associated with BC development [192].

Ferroptosis-associated lncRNAs can serve as prognostic indicators for constructing a prognostic map of BC based on early warning signs, treatment goals and the anti-tumour immune microenvironment of BC to guide clinical therapy. A previous study screened 11 lncRNAs associated with ferroptosis from the TCGA dataset and built a prognostic map. Based on differences in the expression levels of ferroptosis-associated lncRNAs in tissues from BC patients and healthy tissues, patients were grouped into high- and low-risk clusters. In this study, three genes, lncRNAs YTHDF3-AS1, AC079298.3 and AC012213.3, which are overexpressed in at-risk populations, were screened, indicating that they may be at-risk oncogenes for BC. In addition, lncRNA NC012213.3, a downstream molecule of AC012213.3, and overexpressed LncRNA AC012213.3 promotes BC multiplication, invasion and invasiveness through the RAD54B/PI3K/AKT axis and is associated with poor patient outcomes [193]. However, studies on YTHDF3-AS1 and AC079298.3 are limited and require further investigation. To date, lncRNA-associated ferroptosis particularly its association with BC has not been documented, warranting further research to identify new therapeutic targets for BC [190].

## Conclusions

Our study has comprehensively elucidated the underlying mechanism of ferroptosis in relation to BC, providing potential insights for strategising new approaches for anti-tumour therapy. Ferroptosis occurs when there is an imbalance between the detoxification and accumulation of lipid hydroperoxides [194]. A stressful environment can cause an imbalance in lipid ROS accumulation, which contributes to ferroptosis [195]. Cancer cells typically promote survival and metastasis by resisting ferroptosis. Certain drugs used in BC treatment [196], such as cyclophosphamide, tamoxifen, paclitaxel and anthracycline, may induce excessive ROS generation, resulting in cell death [197–200]. LncRNAs are common transcription products in human and mammalian genomes. The roles of certain lncRNAs in tumourigenesis and tumour development have gained considerable attention, with established functions elucidated in primary studies. The disclosure of the universal genetic code has enabled researchers to identify defects in functional proteins; however, understanding the impact of lncRNA biology on cellular function remains challenging using existing predictive frameworks [22]. Owing to their enhanced efficiency, tissue specificity and stability, lncRNAs have the potential to be initial diagnostic and therapeutic targets [190].

Therefore, understanding the relationship between lncRNAs and ferroptosis, as well as their regulatory mechanisms in BC, can be beneficial for the development and therapy of this disease. Our findings demonstrated that lncRNA HCP5 encodes a novel protein, HCP5-132aa, and promotes TNBC growth by controlling GPX4 and inhibiting ROS levels, ultimately inhibiting ferroptosis. Patients with TNBC who overexpress HCP5-132aa typically have worse disease characteristics and prognoses [191]. Ferroptosis-associated lncRNAs may play a prognostic role in BC, enabling the construction of a prognostic model for screening markers, therapeutic targets, evaluating the anti-tumour immune microenvironment and guiding clinical therapy [190]. Nevertheless, future studies investigating the correlation between lncRNAs and ferroptosis and their underlying mechanisms in BC are warranted.

## Data Availability

Not applicable.

## Abbreviations

AA: Arachidonic acid
ACD: Accidental cell death
ACSL4: Acyl-Coenzyme A synthase long chain family member 4
AdA: Adrenaline
AIF: Apoptosis-inducing factor
AIFM2: Apoptosis-inducing factor mitochondria-associated 2
ALOX15: AdA-PE to lipid hydroperoxides by 15-LOX
ART: Artesunate
BC: Breast cancer
BIRC3: Baculoviral IAP repeat containing 3
BLIS: Basal-like immunosuppressive subtype
CAN: Cyclophosphamide
CCTs: Circulating tumor cells
CDH2: Calbindin 2
CDO1: Cysteine dioxygenase 1
CRC: Colorectal cancer
CIP2A-BP: CIP2A-binding peptide
c-Myc: Cellular myeloid cell tumor proto-oncogene
CNA: Combination of cottonin A
CoQ10: Ubiquitin ketone
DHA: Docosahexaenoic acid
DPP4: Dipeptidyl peptidase-4
EGLN1: EgI nine homologue 1
FA: Fatty acid
FINs: Ferroptosis inducers
FOXM1: Forkhead box protein M1
FSP1: Ferrocyte apoptosis suppressor protein 1
FZD7: Frizzled homolog 7
Gas5: Growth inhibitory specific 5
GPX4: Glutathione peroxidase 4
GSH: Glutathione
GSSG: Glutathione disulphide
G3BP1: Ras GTPase-activating protein-binding protein 1
HOTAIR: HOX transcriptional anti-sense intergenic RNAs
HPCAL1: Hippocampalin-like protein 1
HSF-1: Heat-shock factor-1
HSPB1: Heat-shock protein beta 1
IM: Immunomodulatory subtype
Keap1: Keleh-like ECH-associated protein 1
LAR: Luminal androgen receptor subtype
LDHA: Lactate dehydrogenase a
LDL: Low-density lipoprotein
LncRNA: Long non-coding RNA
LLP: Liquid–liquid phase
LOXs: Lipoxygenases
LPO: Lipid peroxide
LPCAT3: Lysophosphatidylcholine acyltransferase 3
LSH: Lymphoid tissue-specific deconjugating enzyme
MES: Mesenchymal-like subtype
MEG3: Maternal expression 3
MUC1-C: Mucin 1C-terminal
MVA: Mevalonate
NCCN: National Comprehensive Cancer Network
Nrf2: Nuclear factor red lineage-associated factor 2
OC: Ovarian cancer
PEITC: Phenethyl isothiocyanate
PL: Piperonylamine
PLOOH: Phospholipid hydroperoxides
PROM2: Prominence protein 2
PTBP1: Polypyrimidine tract-binding protein 1
PUFA: Polyunsaturated fatty acids
RCD: Programmed cell death
ROS: Reactive oxygen species
RRM: RNA recognition motif
RUNX1-IT1: RUNX1 intronic transcript 1
RSL3: GSH peroxidase 4 inhibitor
SLC7A11: Solute carrier family 7 member 11
TGF: Transforming growth factor
TNBC: Triple-negative breast cancer
xCT: Systemic X_C_^−^
ZEB1: Zinc finger E box-binding homology box 1
